# Resistin enhances the expansion of regulatory T cells through modulation of dendritic cells

**DOI:** 10.1186/1471-2172-11-33

**Published:** 2010-06-30

**Authors:** Young Min Son, Sung Min Ahn, Gi Rak Kim, Yang Soo Moon, Sang Hoon Kim, Yeong-Min Park, Woon Kyu Lee, Tae Sun Min, Seung Hyun Han, Cheol-Heui Yun

**Affiliations:** 1 Protein Engineering and Comparative Immunology, Department of Agricultural Biotechnology and Research Institute for Agriculture and Life Sciences, Seoul National University, 599 Gwanak-ro, Gwanak-gu, Seoul 151-921, Republic of Korea; 2 Molecular Biology, Department of Animal Science and Biotechnology, Jinju National University, Jinju 660-758, Republic of Korea; 3 Animal Cell Physiology, Department of Biology, Kyung Hee University, Seoul 130-701, Republic of Korea; 4 Department of Microbiology and Immunology and National Research Laboratory of Dendritic Cell Differentiation and Regulation, Medical Research Institute, Pusan National University, College of Medicine, Busan, Republic of Korea; 5 Center for Advanced Medical Education by BK21 project, College of Medicine, Inha University, Incheon 402-751, Republic of Korea; 6 National Research Foundation of Korea, Daejeon 305-350, Republic of Korea; 7 Department of Oral Microbiology & Immunology, Dental Research Institute, and BK21 Program, School of Dentistry, Seoul National University, Seoul 110-749, Republic of Korea

## Abstract

**Background:**

Resistin, a member of adipokine family, is known to be involved in the modulation of immune responses including inflammatory activity. Interestingly, resistin is secreted by adipocytes in mice and rats whereas it is secreted by leukocytes in humans. However, the mechanism behind the effect of resistin on the expansion of regulatory T cells (Tregs) remains poorly understood. Therefore, we examined regulatory effect of resistin on the induction and cellular modification of Tregs.

**Results:**

Both protein and mRNA expression of *FoxP3*, a representative marker of Tregs, increased in a dose-dependent manner when peripheral blood mononuclear cells were treated with resistin. At the same time, resistin had no direct effect on the induction of *FoxP3 *in CD4^+ ^T cells, suggesting an indirect role through other cells type(s). Since DCs are an important player in the differentiation of T cells, we focused on the role of DCs in the modulation of Tregs by resistin. Resistin suppressed the expression of interferon regulatory factor (IRF)-1 and its target cytokines, IL-6, IL-23p19 and IL-12p40, in DCs. Furthermore, *FoxP3 *expression is increased in CD4^+ ^T cells when co-cultured with DCs and concomitantly treated with resistin.

**Conclusion:**

Our results suggest that resistin induces expansion of functional Tregs only when co-cultured with DCs.

## Background

Resistin, a novel adipocyte-secreted hormone, has gained attention for its involvement in insulin resistance in obesity and diabetes mellitus [1]. In murine animals, resistin is secreted from mature white adipocytes and decreases insulin sensitivity, whereas in humans it is mainly secreted from immune cells such as macrophages and monocytes and organs such as the spleen and bone marrow [2]. Recently, several groups have reported a close relationship between resistin and inflammation. Resistin increases the production of pro-inflammatory cytokines tumor necrosis factor-α (TNF-α) and interleukin (IL)-12, both of which are important for T cell development, through the regulation of NF-κB signaling pathway in human macrophages [3]. In the clinical setting, high levels of resistin expression are present in patients with rheumatoid arthritis [3].

Forkhead box P3 (FoxP3), a key regulator of the development of regulatory T cells (Tregs, CD4^+ ^CD25^+ ^FoxP3^+ ^T cells), is a hallmark of Tregs [4], which exhibit a suppressive response to the effector function of CD4^+ ^T cells [5]. Two types of Tregs have been reported: natural Tregs (nTregs), which naturally occur in the thymus, and inducible Tregs (iTregs), which can be induced from naïve T cells. The iTregs exhibit less suppressive function to effector T cell responses than nTregs [6]. Interleukin (IL)- 2, transforming growth factor-β (TGF-β) and ligands for the activation of T cell receptors (i.e., anti-CD2, -CD3 and -CD28) have been reported as important factors in the induction of Tregs from naïve T cells [7].

Dendritic cells (DCs) play a vital role in the modulation and activation of T cells, not only for the delivery and presentation of antigenic determinant(s) to T cells, but also in the induction of differentiation of naïve T cells into T cells subtypes via cytokine secretion and/or cell-to-cell contact [8]. Recently, several studies have reported that DCs are closely involved in the differentiation of Tregs. For example, DCs either secreting TGF-β or in the presence of TGF-β induce differentiation of CD4^+^CD25^+^FoxP3^+ ^Tregs [9]. Interferon regulatory factor-1 (IRF-1)^-/- ^DCs enhance the suppressive ability of Tregs and cause tolerogenic features via induction of Tregs due to the fact that the IRF-1^-/- ^DCs secrete more IL-10 and TGF-β but less IL-6, IL-12p40 and IFN-γ than wild type DCs [10].

We previously reported that resistin impairs the ability of human monocyte-derived DCs to uptake antigens and produce cytokines (IL-12p40, IL-6 and TNF-α). These impaired DCs negatively regulate T cell responses such as proliferation of and differentiation into Th1 cells [11]. In the current study, we further examine the ability of DCs treated with resistin to induce expansion of Tregs.

## Methods

### Reagents

Human recombinant resistin, endotoxin level less than 0.1 ng/μg, was purchased from Peprotech (Rocky Hill, NJ). To provide the effects of irrelevant control, we transformed recombinant resistin to heat-inactivated resistin through incubation with 100°C for 10 min. RPMI-1640 Glutamax medium, fetal bovine serum (FBS) and antibiotics (100 U/ml penicillin and 100 μg/ml streptomycin) were purchased from Invitrogen (Grand Island, NY).

### Preparation of human monocyte-derived DCs and CD4^+ ^T cells

PBMCs were isolated by density gradient centrifugation (420 × *g* for 25 min without brake) using Ficoll-Paque Plus (Amersham Bioscience, Buckinghamshire, UK). All experiments involving human blood were approved by the Institutional Review Board of Seoul National University (IRB no. 0705/001-002). CD14^+ ^monocytes or CD4^+ ^T cells (purity > 95%) were isolated from PBMCs using magnetic bead-based positive selection with a kit (BD Biosciences, San Diego, CA). CD14^+ ^monocytes were suspended in complete media consisting of RPMI-1640 Glutamax medium supplemented with 10% FBS and 1 mM sodium pyruvate, 100 U/ml penicillin and 100 μg/ml streptomycin. To generate immature DCs, isolated CD14^+ ^monocytes were cultured with 500 U/ml of hrIL-4 and 800 U/ml of hrGM-CSF (both from R & D Systems, Minneapolis, MN) for 6 days with a change of media every 3 days.

### RNA isolation and reverse transcriptase-polymerase chain reaction (RT-PCR)

Cells were lysed with 1 ml of Trizol (Invitrogen) and 200 μl of chloroform was added. The tubes were shaken gently and incubated for 5 min at room temperature. The tubes were centrifuged at 12,000 × *g* for 17 min and the aqueous phase containing RNA was transferred into a new tube. After transfer, 0.5 ml of isopropanol was added to the RNA solution, mixed by gentle inversion, and then incubated for 10 min at room temperature. The suspended RNA was precipitated by centrifugation at 12,000 × *g* for 12 min. The supernatant was discarded and the RNA pellet was washed with 75% ethanol followed by centrifugation at 7500 × *g* for 7 min. The supernatant was discarded and the RNA pellet was re-suspended in nuclease-free water.

Isolated total RNA was reverse-transcribed into cDNA with oligo-dT primers (Promega, San Luis Obispo, CA). The cDNA was amplified by PCR in a total volume of 10 μl containing 0.5 units of *Taq *polymerase and two picomoles of specific primers for human FoxP3, TGF-β, IRF-1, IL-12p40, IL-6, IL-10, CTLA-4 or β-actin (Table 1). PCR was performed in a thermal cycler (Bio-Rad Laboratories, Hercules, CA) with pre-denaturation at 94°C for 5 min, followed by 28 cycles of denaturation at 94°C for 30 sec, annealing at the appropriate annealing temperature (Table 1) for 30 sec and extension at 72°C for 1 min, and finished with an additional elongation step for 7 min at 72°C. Amplified PCR products were separated by electrophoresis using a 2% agarose gel containing 1 μg/ml ethidium bromide. To quantitate the expression of each mRNA, the ratio of each mRNA to β-actin was measured using Multi-gauge software (Fujifilm, Tokyo, Japan).

**Table 1:** The primers used in the present study for RT-PCR

Primer	Sequences	AT*	Product size	NCBI numbers
		(°C)	(bp)	
*FoxP3*	forward: 5'-AGT TCC TCC ACA ACA TGG AC-3'	57	219	NM_014009
	reverse: 5'-CAA AGC ACT TGT GCA GAC TC-3'			
*TGF-β*	forward: 5'-AAA GAC TTT TCC CCA GAC CT -3'	58	155	NM_000660
	reverse: 5'-AAG GTG GGT GGT CTT GAA TA -3'			
*IRF-1*	forward: 5'-GTG AAA GAC CAG AGC AGG-3'	57	400	NM_002198
	reverse: 5'-CTG TTG TAG CTT CAG AGG-3'			
*IL-12p40*	forward: 5'- GGA CCA GAG CAG TGA GGT CTT-3'	57	209	AF180563
	reverse: 5'-CTC CTT GTT GTC CCC TCT GA-3'			
*IL-6*	forward: 5'- TAG CCG CCC CAC ACA GAC AG-3'	61	408	NM_000600
	reverse: 5'-GGC TGG CAT TTG TGG TTG GG-3'			
*IL-10*	forward: 5'- AGA AAG GCA TCT ACA AAG CCA-3'	58	239	NM_000572
	reverse: 5'-TGG GGG TTG AGG TAT CAG AG-3'			
*CTLA-4*	forward: 5'- AGT TTT GTG GAG GAG CTC AG-3'	57	247	AF414120
	reverse: 5'-CTC CTG GAG TAA GCC ATT GT-3'			
*β-actin*	forward: 5'- GGA CTT CGA GCA AGA GAT GG-3'	55	234	NM_001101
	reverse: 5'-AGC ACT GTG TTG GCG TAC AG-3'			

*AT, annealing temperature

### IRF-1 detection by Western blot analysis

5 × 10^5 ^cells of DCs incubated with 500 ng/ml of resistin for different time periods (0, 1, 6, 12 and 24 hrs) in 24-well plate (Nalgene Nunc International, Rochester, NY) were washed with cold phosphate buffered saline (PBS) and then lysed in RIPA lysis buffer containing 150 mM NaCl, 1% NP-40, 0.5% deoxycholate, 0.1% SDS, 50 mM Tris pH7.4, protease inhibitor cocktail (Roche, Mannheim, Germany), 2 mM NaF, 0.1 mM sodium orthovanadate and 2 mM glycerol phosphate. After centrifugation at 28,000 × *g* for 7 min at 4°C, the supernatant was transferred to a new tube and the concentration of protein was determined by Bradford assay (Bio-Rad Laboratories) with bovine serum albumin (BSA) as a standard. Twenty-five micrograms of proteins were separated by 12% SDS-polyacrylamide gel electrophoresis and transferred to a polyvinylidene difluoride membrane (Amersham Bioscience). The membrane was blocked with 5% non-fat milk containing Tris-buffered saline Tween-20 (TBST; 0.1 M Tris, 0.9% NaCl, and 0.1% Tween 20) overnight at 4°C. After washing three times with TBST, the membrane was incubated with rabbit anti-human IRF-1 antibody (Santa Cruz Biotechnology, Inc) for 3 hrs at room temperature and washed with TBST three times. Protein bands were detected using mouse anti-rabbit IgG conjugated with horseradish peroxidase (Chemicon, Temecula, CA) and developed by the enhanced chemiluminescence system (GE Healthcare, Buckinghamshire, UK).

### TGF-β detection by enzyme-linked immunosorbent assay (ELISA)

CD4^+ ^T cells and DCs (2×10^5 ^cells each) were co-cultured with or without 0.5 μg/ml of resistin for 4 days. Anti-human CD2 and CD3 antibodies (0.1 μg/ml each) (Miltenyi Biotec, Auburn, CA) were then added and the cultures were incubated for 3 more days. The concentration of human TGF-β in the supernatant was measured using an ELISA DuoSet kit (R & D Systems). Briefly, TGF-β capture antibody was coated on each well of a 96-well plate (Nalgene Nunc International) and incubated overnight at 4°C. The wells were blocked with blocking buffer (0.1% BSA in PBS) for 1 hr. After oxidation-reduction of the supernatant to activate TGF-β, the supernatants from the culture or standard samples were added and incubated for 2 hrs at room temperature. Next, detection antibody conjugated with biotin was added to each well and incubated for 2 hrs at room temperature. The plates were washed three times with washing buffer (0.05% Tween 20 in PBS) between each step. The specific reaction was detected using streptavidin-HRP followed by TMB in the substrate buffer (Sigma-Aldrich Co., Saint Louis, MO). The reaction was stopped with 2NH_2_SO_4 _and the amount of TGF-β was measured by microplate reader (Molecular Devices, Sunnyvale, CA).

### Flow cytometric analysis

Cells cultured under different conditions were harvested and washed three times with cold PBS. The cells were stained with the desired combination of anti-human CD25-APC and FoxP3-PE antibodies (BD biosciences) for 20 min at 4°C in the dark. The cells were washed and changes in marker expression were measured using a FACSCalibur with Cell-Quest software (BD Biosciences). All flow cytometric data were analyzed with FlowJo software (Tree Star, San Carlos, CA).

To evaluate the effect of TGF-β on the induction of Tregs, CD4^+ ^T cells co-cultured with DCs were pre-treated for 1 hr TGF-β receptor I inhibitor (Calbiochem, San Diego, CA). After pre-treatment, the cells were incubated with or without resistin (0.5 μg/ml) for 4 days and then anti-human CD2 and CD3 antibodies were added and the cultures were incubated for an additional 3 days. After staining the cells with anti-human CD25-APC and FoxP3-PE antibodies, CD25^+ ^FoxP3^+ ^Tregs were detected using flow cytometry as described above.

### Suppression activity of Tregs against CD4^+ ^CD25^- ^T cells

CD4^+ ^T cells and DCs were co-cultured with or without 0.5 μg/ml of resistin for 4 days and then anti-human CD2 and CD3 antibodies (Miltenyi Biotec) were added and the cultures were incubated for 3 more days. After washing with cold PBS, the cells were stained with anti-human CD25-APC antibody for 20 min at 4°C in the dark and then washed with cold PBS. CD25^+ ^T (e.g., CD4^+ ^CD25^+ ^) effector cells (purity > 97%) were isolated using anti-APC particles (BD biosciences) together with IMag™, a magnetic bead-based positive selection kit (BD biosciences). CD4^+ ^CD25^- ^T cells (purity > 95%) were isolated using a regulatory T cell isolation kit (Miltenyi Biotec), labeled with 2.5 μM of CFSE (Invitrogen) for 8 min in 37°C and used as target cells. Effector cells (2×10^5 ^cells) and an equal number of target cells were co-cultured with anti-human CD2 and CD3 antibodies (Miltenyi Biotec) for an additional 5 days in a 24-wells plate. The intensity of CFSE in the target cells was measured using a FACSCalibur with CellQuest (BD Biosciences). All flow cytometric data were analyzed by FlowJo software (Tree Star).

### Statistical analysis

Comparative data were analyzed using Student's *t*-test and considered statistically significant when the *P *value was less than 0.05.

## Results

### Resistin induces expression of *FoxP3 *in PBMC, but not in CD4^+ ^T cells

**Figure 1:**
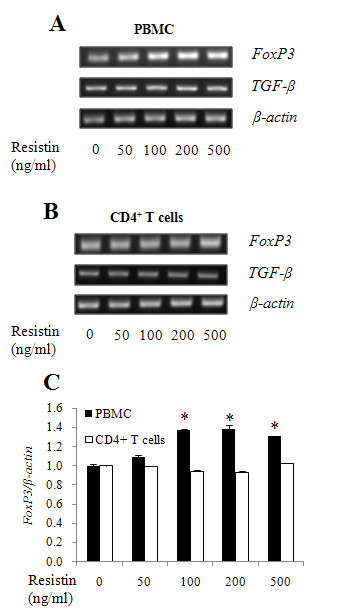
**Expression of *FoxP3 *mRNA in PBMCs or CD4^+ ^T cells treated with resistin**. (A) PBMC and (B) CD4^+ ^T cells isolated from human adult peripheral blood were treated with various concentrations of resistin (0, 50, 100, 200 or 500 ng/ml) for 6 hrs. For measurement of *FoxP3 *and *TGF-β *mRNA expression, RT-PCR was performed as described in Materials and Methods. (C) The intensities of the *FoxP3 *were normalized to β-actin. Results are representative of three separate experiments with similar results.

To examine whether resistin could modulate the induction of Tregs, we tested the expression level of *FoxP3 *in PBMCs and CD4^+ ^T cells. FoxP3 is one of the most representative markers of Tregs, together with CTLA4, TGF-β and IL-10 [6]. Since FoxP3 is expressed in T cells only, PBMCs and CD4^+ ^T cells were incubated with 0, 50, 100, 200 or 500 ng/ml of resistin for 6 hrs. The expression level of *FoxP3 *was then measured by RT-PCR. The expression of *FoxP3 *increased significantly (*P*< 0.05) in a dose-dependent manner in PBMCs treated with resistin (figure 1A and 1C). Minimal, if any, change was found in the expression of *TGF-β *(figure 1A). It is to note that the expression level of *FoxP3 *and *TGF-β *was, unexpectedly, not changed in CD4^+ ^T cells treated with any concentration of resistin (figure 1B and 1C). These results suggest that the resistin-induced expression of *FoxP3 *in Tregs is mediated by indirect where CD4^+ ^cells are not necessarily required.

### Resistin-treated DCs enhance the expansion of Tregs

**Figure 2:**
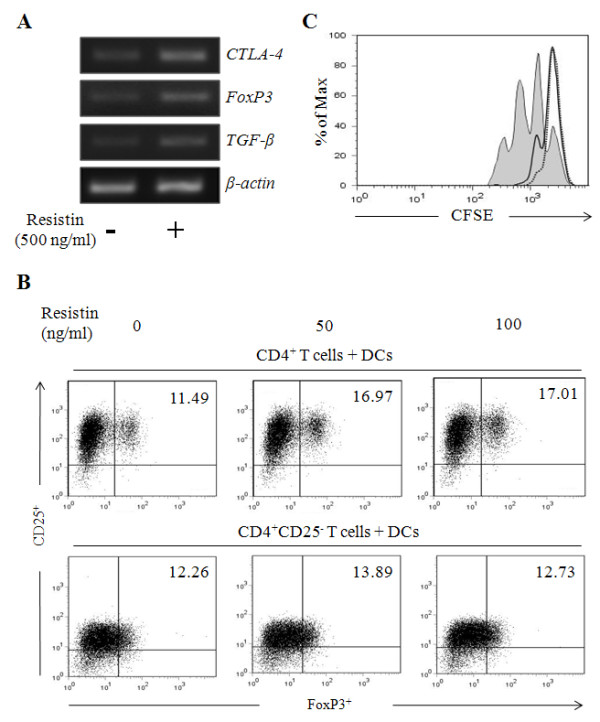
**The number of Tregs increased when co-cultured with DCs and concomitantly treated with resistin**. CD4^+ ^T cells or CD4^+ ^CD25^- ^T cells and DCs were co-cultured with 500 ng/ml of resistin for 4 days. The cells were then incubated with anti-human CD2 and CD3 antibodies for an additional 3 days. (A) At the end of the incubation period, total RNA was isolated and subjected to RT-PCR to measure mRNA expression of *CTLA-4*, *FoxP3 *and *TGF-β*. (B) After the staining of the cells with anti-human CD25-APC and -FoxP3-PE antibodies, CD25^+^FoxP3^+ ^Tregs were analyzed by flow cytometry. (C) After the staining with anti-human CD25-APC antibody, CD25^+ ^T cells were isolated using anti-APC magnetic beads. The isolated CD25^+ ^T cells and CD4^+ ^CD25^- ^naïve T cells labeled with CFSE were co-cultured with DCs and stimulated with anti-human CD2 and CD3 antibodies for 5 days. Cell proliferation was measured by flow cytometry. The value in each panel indicates the percentage of CD25^+ ^FoxP3^+ ^Tregs.

The primary role of DCs is to interact with T cells and trigger adaptive immune responses, but DCs are also involved in the differentiation of Tregs [12]. Therefore, to further examine whether resistin-treated DCs are involved in the regulation of Tregs, CD4^+ ^T cells and DCs were co-cultured and concomitantly treated with resistin. mRNA expression levels of *TGF-β*, *CTLA-4 *and *FoxP3*, representative markers of Tregs, were subsequently measured. The expression of *TGF-β*, *CTLA-4 *and *FoxP3 *was greater in cells treated with resistin than those without resistin treatment (figure 2A). This finding was further confirmed by changes of CD4^+ ^CD25^+ ^FoxP3^+ ^Tregs using flow cytometry. As shown in figure 2B (upper panel), CD4^+ ^CD25^+ ^FoxP3^+ ^cells were significantly (*P *< 0.05) higher in cells treated with resistin than those without treatment (additional file 1). And, to confirm whether the increased number of Tregs were induced from naïve CD4^+ ^T cells or expanded from Tregs, CD4^+ ^CD25^- ^naïve T cells were co-cultured and concomitantly treated with resistin. The result showed that CD25^+ ^FoxP3^+ ^Tregs were expanded from Tregs but were merely induced from CD4^+ ^CD25^- ^naïve T cells when co-cultured with resistin-treated DCs (figure 2B bottom panel). Finally, CD4^+ ^CD25^+ ^T cells were isolated after co-culture of T cells and DCs concomitantly treated with/without resistin in order to examine their functional capacity to suppress the activity of effector T cells. CD4^+ ^CD25^- ^T cells labeled with CFSE were co-cultured with CD4^+ ^CD25^+ ^T cells and stimulated with anti-human CD2 and CD3 antibodies. When compared with a proliferation of CD4^+ ^CD25^- ^naïve T cells only, the CD4^+ ^CD25^+ ^Tregs induced by DCs treated with resistin suppressed the proliferation of CD4^+ ^CD25^- ^naïve T cells similar to CD4^+ ^CD25^+ ^T cells induced by DCs without resistin (figure 2C). These results suggest that resistin modulates the functionality of DCs to enhance the expansion of CD4^+ ^CD25^+ ^FoxP3^+ ^Tregs.

### The effects of resistin in DCs for expansion of Tregs

**Figure 3:**
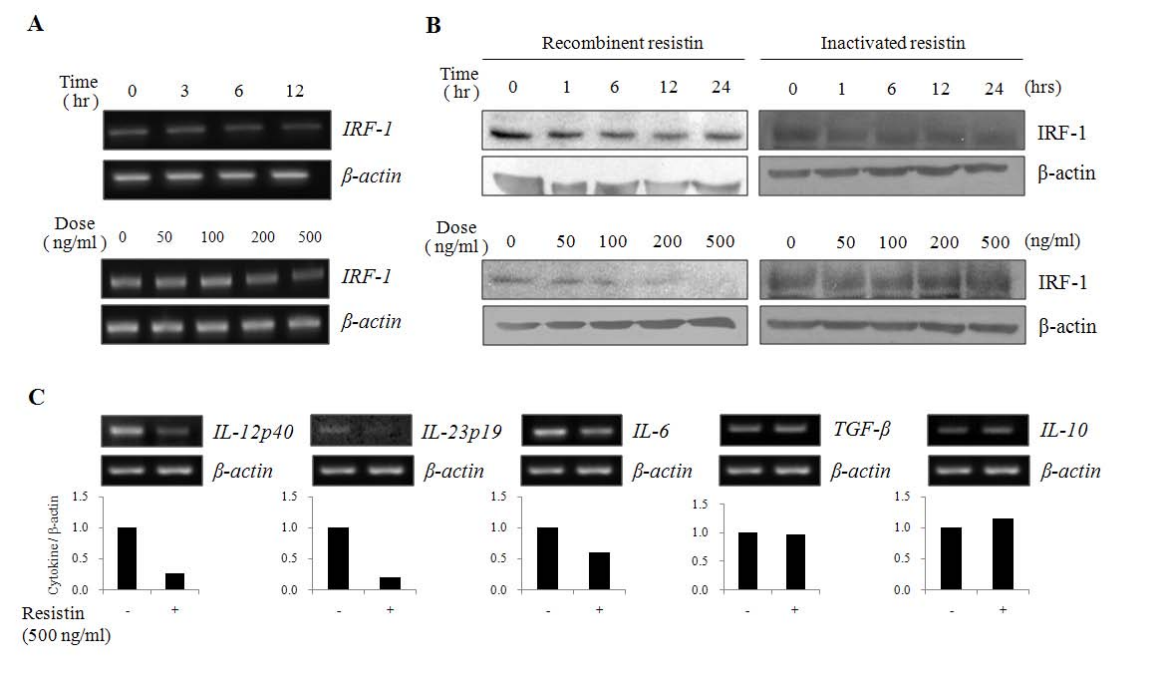
**Resistin suppressed the expression of IRF-1 and cytokines in DCs**. The expression of IRF-1, in both (A) mRNA and (B) protein was determined in DCs treated with 500 ng/ml of resistin for the indicated time periods (upper panel in A and B) and treated with indicated amount for 24 hrs (bottom panel in A and B). The heat-inactivated resistin was used as a control. (C) The expression of *IL-12p40*, IL-23p19, *IL-6*, *TGF-β *and *IL-10 *was measured by RT-PCR in DCs treated with 500 ng/ml of resistin for 12 hrs. The graphs show the intensity of each cytokine normalized to β-actin. Results are representative of three separate experiments with similar result.

Next, we examined the change of IRF-1 and cytokines, IL-12p40, IL-23p19, IL-6, IL-10 and TGF-β in relation to the differentiation of T cells. IRF-1 both, at mRNA (figure 3A) and protein level (figure 3B) was suppressed in a time- and dose-dependent manner when DCs were treated with resistin.  Note that heat-inactivated resistin was used. It has been previously shown that the expression of inflammatory cytokines, *TNF-α*, *IFN-γ*, *IL-12p40 *and *IL-15*, was significantly reduced while the expression of tolerogenic cytokines, *IL-10 *and *TGF-β*, was increased in DCs from IRF^-/- ^mice [10]. IL-12, IL-23 and IL-6 are known to be involved in the differentiation of naïve T cells into Th1 and Th17 subtypes, respectively, while IL-10 and TGF-β induce the differentiation of Tr1 and Th3 subtypes, respectively [13]. Accordingly, we further examined the expression of *IL-12p40*, *IL-23p19*, *IL-6*, *IL-10 *and *TGF-β *in DCs after resistin treatment. The expression of *IL-12p40, IL-23p19 *and *IL-6 *decreased in DCs treated with resistin, while no significant differences were observed in the expression of *IL-10 *and *TGF-β *(figure 3C). And through the PI/annexin V, CD40, CD80, CD86 and MHC class II staining, we confirmed that resistin is involved in the process of neither apoptosis nor maturation of DCs (data not shown). These results suggest that resistin suppresses the expression of *IL-12p40, IL-23p19 *and *IL-6 *through the regulation of IRF-1 induction in DCs.

### Tregs were induced by TGF-β secreted by themselves but not by DCs treated with resistin

**Figure 4:**
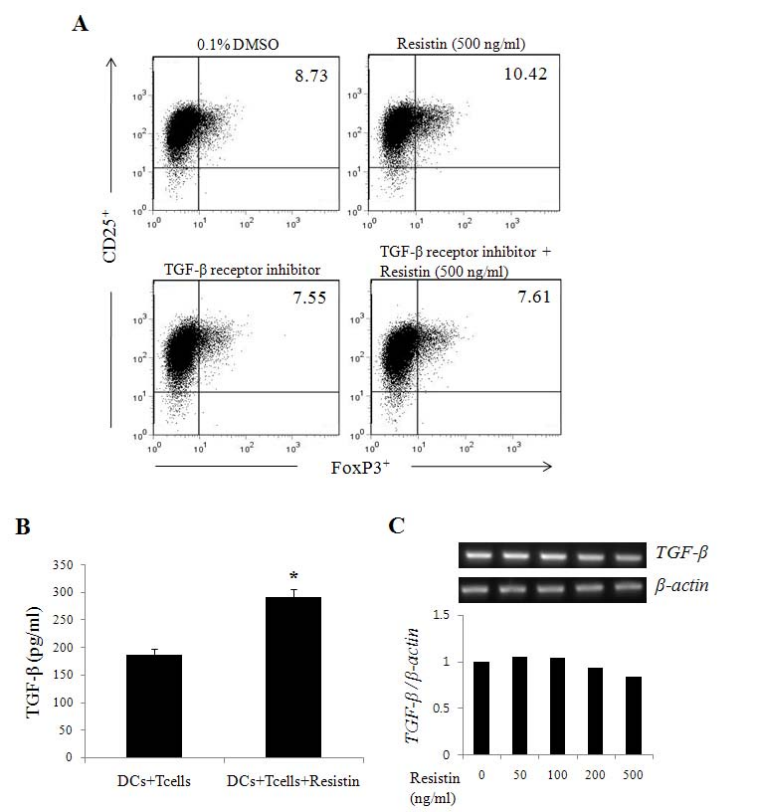
**TGF-β secreted by CD4^+ ^CD25^+ ^FoxP3^+ ^Tregs, but not by DCs, induces the expansion of Tregs**. CD4^+ ^T cells were pre-treated with inhibitor for TGF-β receptor for 1 hr and co-cultured with DCs, followed by resistin treatment for 4 days. Anti-human CD2 and CD3 antibodies were then added to the cells for an additional 3 days. (A) CD25^+ ^FoxP3^+ ^Tregs were measured by flow cytometry. (B) TGF-β production in the supernatant was measured by ELISA. (C) DCs were treated with various concentrations (0, 50, 100, 200 and 500 ng/ml) of resistin for 6 hrs and then *TGF-β *mRNA expression was measured by RT-PCR. * *P*< 0.05. The value in each panel indicates the percentage of CD25^+ ^FoxP3^+ ^Tregs. The results represent the mean ± standard deviation from three separate experiments.

To investigate whether TGF-β secreted from DCs is involved in the induction of Tregs, CD4^+ ^T cells were pre-treated with TGF-β receptor inhibitor, co-cultured with DCs, and concomitantly treated with resistin. Tregs were induced when the cells were treated with resistin as expected, but treatment with TGF-β receptor inhibitor suppressed such induction (figure 4A). We also examined the level of TGF-β in the culture supernatant. TGF-β increased in CD4^+ ^T cells when co-cultured with DCs and treated with resistin (figure 4B). The expression of *TGF-β *mRNA was not significantly different in DCs alone treated with resistin (figure 4C). These results suggest that TGF-β produced from CD4^+ ^T cells co-cultured with DCs together with resistin treatment, was the major source to induce the differentiation of Tregs, rather than TGF-β secreted from DCs.

## Discussion

The major findings of this study are as follow: (i) Resistin indirectly induced the expression of *FoxP3 *in PBMCs, (ii) Resistin suppressed the expression of IRF-1, IL-6, IL-12p40 and IL-23p19 in DCs, (iii) DCs treated with resistin enhanced the expansion of CD4^+ ^CD25^+ ^FoxP3^+ ^T cells, (iv) TGF-β caused the expansion of Tregs and (v) TGF-β was mainly produced from Tregs during the co-culture of T cells and DCs together with resistin treatment.

Resistin was originally described because of its promotion of resistance against the insulin response [1] and adipogenesis in fat tissue [14]. More recently, important roles of resistin in the human immune response [15] and inflammation, including induction of pro-inflammatory cytokines, [2,3,16] have been reported. In the previous study, we reported the immunomodulatory effect of resistin on human DCs for the first time [11]. We have demonstrated that resistin impairs not only the ability of antigen-uptake and cytokine production in DCs, but also the proliferation and differentiation of T cells. These results suggest that the DCs treated with resistin may negatively regulate the effector function of T cells. Our findings are in agreement with those of Rutella *et al*., who also reported that tolerogenic DCs, known to express high amounts of IL-10 but not IL-12 in the immature state, induce Tregs [17]. It is to note that Tregs are well known for their role in suppressing a variety of T cell responses. Tregs also regulate DCs and NK cells by suppressing expression of the CCR5 ligand and IL-15Rα, respectively [18]. Accordingly, we hypothesized that resistin could be involved in the expansion of Tregs through the regulation of DCs.

In order to clarify whether resistin could induce the expansion of Tregs, we treated PBMCs and purified CD4^+ ^T cells with resistin and analyzed the changes in *FoxP3* expression. Resistin had no direct effect on the induction of *FoxP3 *mRNA expression in CD4^+ ^T cells, but did increase *FoxP3 *mRNA in PBMCs. This result suggests that resistin may indirectly induce Tregs through the regulation of other cell types, most likely DCs. Tregs can be induced by cell-to-cell contact, for instance, DCs and T cells. DCs, producing TGF-β, IL-10 and indoleamine 2,3-dioxygenase can induce the differentiation of Tregs *in vitro *and *in vivo *[12]. Since immature DCs have a better ability to induce Tregs than mature DCs [19], we examined changes in the expressin of DC maturation markers (CD40, CD80, CD86 and HLA-DR) following resistin treatment (data not shown). We found no evidence for the involvement of resistin in the maturation of DCs, implying that the induction of Tregs is independent of DC maturation.

We further demonstrated that resistin suppressed the expression of IRF-1 and cytokines such as IL-6, IL-12p40 and IL-23p19 in DCs, suggesting the possibility that resistin-treated DCs are involved in the induction of Tregs. Indeed, IL-6 enhances the conversion of Th17 cells to Tregs when combined with TGF-β, whereas it inhibits leukemia inhibitory factor (LIF), which is an essential factor for inducing FoxP3 expression in Tregs [20]. On the other hand, IL-12 is known as one of the most important cytokines for inducing differentiation of naïve T cells into Th1 cells [21]. IL-23p19 is well known as the important source to induce Th17 cells [13]. Therefore, these decrease of effect cytokines support the enhancing expansion of Tregs.

The down-regulation of IRF in our findings is supported by a previous study in IRF-1^-/- ^mice, which have high levels of CD4^+^CD25^+^FoxP3^+ ^Tregs, where the expression of FoxP3 was negatively regulated by IRF-1 [22]. We found that the expression of FoxP3 and CTLA-4 increased in parallel with the suppressive effect of Tregs when CD4^+ ^T cells were co-cultured with DCs in the presence of resistin.

It has been shown that suppressors of cytokine signaling (SOCS) 3-deficient DCs, together with impaired production of cytokines (IL-12, IFN-γ and IL-6) and co-stimulatory molecules (CD40 and CD86), preferentially induce the expansion of Tregs [23]. Our results also suggest that resistin decreases the production of SOCS3 in DCs (data not shown). Tregs can be induced by soluble factors, including TGF-β, IL10 and IL-2. TGF-β, in particular, suppresses inflammatory activity and promotes differentiation of Tregs [5]. Accordingly, we examined the source of TGF-β that provoked the expansion of Tregs. We found that major source of TGF-β required for the induction of Tregs was CD4^+ ^T cells co-cultured with DCs in the presence of resistin, and not DCs themselves treated with resistin. It is to note that the treatment with TGF-β or supernatant from any culture condition aforementioned had no impact on the expansion of Tregs (data not shown). Therefore it is most likely that in addition to TGF-β produced from co-culture of CD4^+ ^T cells and DCs in the presence of resistin, cell to-cell contact is critically required for the induction of Tregs.

## Conclusions

We have shown that resistin induces the expansion of regulatory T cells through regulating the expression of IRF-1 and its target cytokines, IL-6, IL-23p19 and IL-12p40 in human monocyte-derived dendritic cells.

## Abbreviations

BSA: bovine serum albumin; CFSE: carboxyfluorescein succinimidyl ester; CTLA: cytotoxic T-lymphocyte antigen; DCs: dendritic cells; FBS: fetal bovine serum; FoxP3: forkhead box P3; GM-CSF: granulocyte macrophage colony-stimulating factor; HRP: horseradish peroxidase; IL: interleukin; IRF: interferon regulatory factor; PBMC: peripheral blood mononuclear cells; PBS: phosphate buffered saline; PI: propidium iodide; SOCS: suppressors of cytokine signaling; TBST: Tris-buffered saline Tween-20; TGF: transforming growth factor-β; TMB: 3,3',5,5'-tetramethylbenzidine dihydrochloride; TNF: tumor necrosis factor; Tregs: regulatory T cells; nTregs: natural Tregs; iTregs: inducible Tregs.

## Authors' contributions

CHY and SHH conceived of and designed the study. YMS, SMA and GRK performed most RT-PCR, western blot and flow cytometry analyses. YMS drafted the manuscript. YSM and SHK advised designing the current experiment and editing the manuscript. YMP, WKL and TSM participated in the statistical analysis, its coordination and discussion of the manuscript. All authors have read and approved the final manuscript.

## Supplementary Material

Additional file 1 **Resistin-treated DCs quantitatively enhance the expansion of CD4^+^CD25^+ ^FoxP3^+ ^Tregs**. CD4^+ ^T cells and DCs were co-cultured with 0, 50 or 100 ng/ml of resistin for 4 days. The cells were then incubated with anti-human CD2 and CD3 antibodies for an additional 3 days. After the staining of the cells with anti-human CD25-APC and FoxP3-PE antibodies, CD25^+ ^FoxP3^+ ^Tregs were analyzed by using flow cytometry. The quantitative frequency of CD25^+ ^FoxP3^+ ^Tregs was plotted with five independent experiments. * indicates significant difference at *P *< 0.05 compared to no resistin treated group. Click here for file
