# Supplementary figures and images for: Resistin enhances the expansion of regulatory T cells through modulation of dendritic cells
Source: BMC Immunol. 2010 Jun 30;11:33. doi: 10.1186/1471-2172-11-33 (PMC2914082; doi:10.1186/1471-2172-11-33)

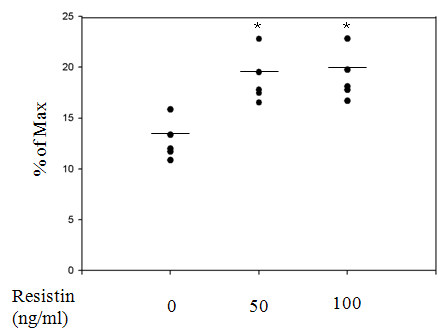

Supplement: Additional file 1 — Resistin-treated DCs quantitatively enhance the expansion of CD4+CD25+ FoxP3+ Tregs. CD4+ T cells and DCs were co-cultured with 0, 50 or 100 ng/ml of resistin for 4 days. The cells were then incubated with anti-human CD2 and CD3 antibodies for an additional 3 days. After the staining of the cells with anti-human CD25-APC and FoxP3-PE antibodies, CD25+ FoxP3+ Tregs were analyzed by using flow cytometry. The quantitative frequency of CD25+ FoxP3+ Tregs was plotted with five independent experiments. * indicates significant difference at P < 0.05 compared to no resistin treated group. [file 1471-2172-11-33-S1.TIFF]
